# Subcellular distribution of nuclear import-defective isoforms of the promyelocytic leukemia protein

**DOI:** 10.1186/1471-2199-11-89

**Published:** 2010-11-21

**Authors:** Åsne Jul-Larsen, Amra Grudic, Rolf Bjerkvig, Stig O Bøe

**Affiliations:** 1 Department of Biomedicine, University of Bergen, Jonas Lies Vei 91, 5009 Bergen, Norway; 2 Norlux Neuro - Oncology, Centre Recherche Public Sante Luxembourg, Luxembourg; 3 Centre for Molecular Biology and Neuroscience (CMBN), Rikshospitalet, Songsvannsveien 20, 0027 Oslo, Norway; 4 Institute of Clinical Biochemistry, University of Oslo, Songsvannsveien 20, 0027 Oslo, Norway

## Abstract

**Background:**

The promyelocytic leukemia (PML) protein participates in a number of cellular processes, including transcription regulation, apoptosis, differentiation, virus defense and genome maintenance. This protein is structurally organized into a tripartite motif (TRIM) at its N-terminus, a nuclear localization signal (NLS) at its central region and a C-terminus that varies between alternatively spliced isoforms. Most PML splice variants target the nucleus where they define sub-nuclear compartments termed PML nuclear bodies (PML NBs). However, PML variants that lack the NLS are also expressed, suggesting the existence of PML isoforms with cytoplasmic functions. In the present study we expressed PML isoforms with a mutated NLS in U2OS cells to identify potential cytoplasmic compartments targeted by this protein.

**Results:**

Expression of NLS mutated PML isoforms in U2OS cells revealed that PML I targets early endosomes, PML II targets the inner nuclear membrane (partially due to an extra NLS at its C-terminus), and PML III, IV and V target late endosomes/lysosomes. Clustering of PML at all of these subcellular locations depended on a functional TRIM domain.

**Conclusions:**

This study demonstrates the capacity of PML to form macromolecular protein assemblies at several different subcellular sites. Further, it emphasizes a role of the variable C-terminus in subcellular target selection and a general role of the N-terminal TRIM domain in promoting protein clustering.

## Background

The PML protein participates in several different cellular functions, including transcription regulation, differentiation, virus defence and tumour suppression [1-4]. In addition, this protein represents one of the two fusion partners in the PML/retinoic acid receptor alpha (RARA) fusion oncoprotein, which supports tumorigenesis in patients with acute promyelocytic leukemia [5,6].

PML belongs to a group of more than 70 different human proteins commonly referred to as the TRIM family of proteins. These proteins are characterized by the presence of a tripartite motif (TRIM) at their N-terminus, which generally comprises three different structural elements, including a RING domain, one or two B-boxes and a coiled coil. The C-terminal region of these proteins typically contains different types of functional domains and may vary between protein isoforms due to alternative pre-mRNA splicing [7,8]. Some common functions of TRIM family members have been identified. For example, a number of members appear to function in the innate immune defence against viruses and several have been shown to possess ubiquitin ligase activity [7,9,10]. In addition, TRIM family proteins appear to have a general propensity to form macromolecular protein assemblies at various subcellular compartments [8,11,12]. It is not clear, however, how the conserved structural organization of TRIM family members contributes to these functions at the molecular level.

A unique feature of the PML protein is its ability to support the structural integrity of nuclear compartments called PML nuclear bodies (PML NBs). These structures can readily be detected by immunofluorescence microscopy as numerous foci within the nucleus, and they recruit a multitude of different proteins with diverse cellular functions [1]. The ability of PML to induce the formation of these structures is facilitated by the TRIM domain, SUMO conjugated residues and a SUMO interacting motif [13-15].

The PML protein is expressed as several alternatively spliced isoforms, and a selected group of these have been designated PML I through PML VII [16,17]. The PML splice variants identified to date contain identical N-termini, including the TRIM domain, whereas the C-termini vary considerably among different isoforms. It is therefore likely that the N-terminus performs a function that is shared by the different isoforms and that the C -terminal variable domain contributes to isoform-specific functions. In agreement with this, some isoform-specific functions of PML have been identified [18-21]. The variability of the PML C-termini probably contributes significantly to the ability of PML to participate in a large variety of different cellular processes.

Although most PML isoforms target PML NBs, splice variants lacking the central nuclear localization signal (herein referred to as NLS6 because it originates from exon 6 of the PML pre-mRNA) have also been identified [22] and may therefore have cytoplasmic functions [16,23,24]. In addition, the PML I isoform is known to contain a nuclear export domain at its variable C-terminus, suggesting that it may shuttle between the nucleus and the cytoplasm [25]. To identify potential cytoplasmic PML targets, we have analysed the subcellular distribution of different PML isoforms containing a mutated NLS6 in the osteosarcoma cell line U2OS. Our analyses show that PML has the potential to target different subcellular compartments beside PML NBs, including early endosomes, late endosomes/lysosomes and the inner nuclear membrane. Subcellular targeting by PML is determined by the isoform specific C-terminal sequence as well as by the presence or absence of a functional NLS6. In addition, the PML TRIM domain is found to have a general role in protein clustering at each of the different target compartments.

## Results

### Differential compartment targeting of import-defective PML isoforms

**Figure 1:**
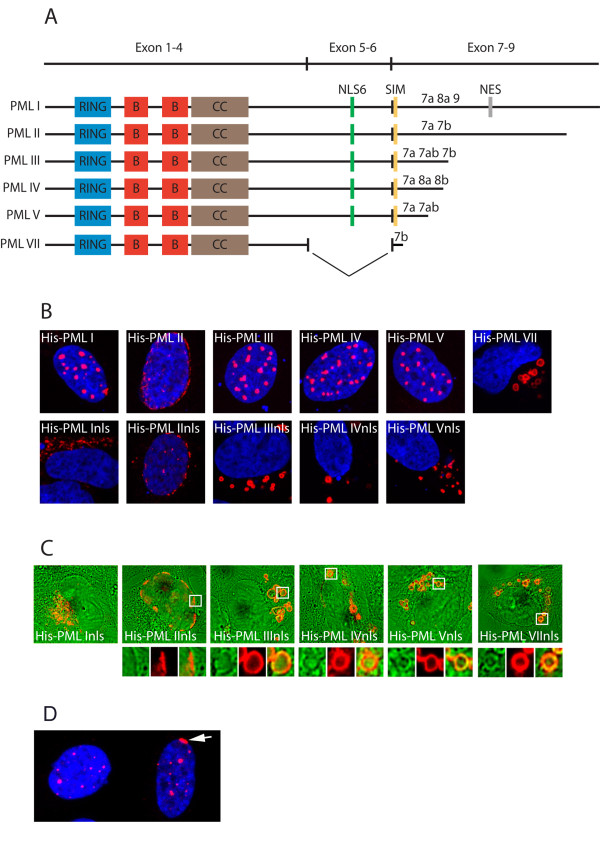
**Subcellular localization of NLS6-defective PML isoforms**. A) Schematic of PML isoforms analyzed in the present study. Regions containing the RING domain (RING), B-boxes (B), Coiled-coil (CC), the nuclear localization signal encoded by exon 6 (NLS6), the SUMO interaction motif (SIM) and the PML I-specific nuclear export signal (NES) are shown. B) Representative images showing subcellular localization of His-tagged PML isoforms with or without a functional NLS6. His-tagged PML was visualized using anti-His antibodies. C) Overlay images showing fluorescently labelled His-tagged isoform (red) and phase contrast images (green). Magnifications show co-localization of PML and membrane enclosed vesicles. D) Endogenous PML in U2OS cells. Arrow points to PML at the nuclear periphery.

Previous studies have identified NLS6 as the primary NLS of the PML protein and PML isoforms encoded by mRNAs lacking exon 6 are generally thought to have lost their nuclear import capacity [16,22]. To generate PML isoforms that are prohibited from entering the nucleus, we mutated two lysines at position 486 and 487 within NLS6 to alanines in five different PML isoforms (PML I through PML V; Figure 1A). Transient expression of His-tagged versions of these mutated proteins, which we designated PML Inls through PML Vnls, in U2OS cells revealed variations in subcellular distribution between different PML isoforms (Figure 1B). PML Inls was mainly detected as an amorphous staining pattern at the peri-centriolar region of the cytoplasm, a region of the cell that contains different types of organelles, including the trans-golgi apparatus, the endoplasmatic reticulum, the microtubules organising centre and mitochondria (Figure 1B). PML IInls, on the other hand, was primarily detected at the nuclear periphery. In fact, the localization of PML IInls appeared to be identical to that of the wild type PML II protein, indicating that this PML splice variant is unaffected by mutations that disrupt NLS6 (Figure 1B). Finally, PML IIInls, PML IVnls and PML Vnls were all found to target circular structures in the cytoplasm suggestive of large membrane embedded vesicles. Interestingly, these cytoplasmic compartments were reminiscent of those targeted by overexpressed PML VII, which represents a naturally occurring cytoplasmic isoform lacking NLS6 (Figure 1B). By overlying the fluorescence images of these circular structures with phase contrast images, we confirmed that the cytoplasmic compartments targeted by PML VII and import-defective PML III, IV and V represented membrane embedded cytoplasmic vesicles (Figure 1C). Analysis of endogenous PML in U2OS cells revealed distribution of this protein mainly to PML NBs. Partial localization of PML to the nuclear periphery could be seen in approximately 3% of cells, whereas the presence of PML at sites of early or late endosomes was generally undetectable (Figure 1D).

### Targeting of early endosomes and late endosomes/lysosomes by cytoplasmic PML

**Figure 2:**
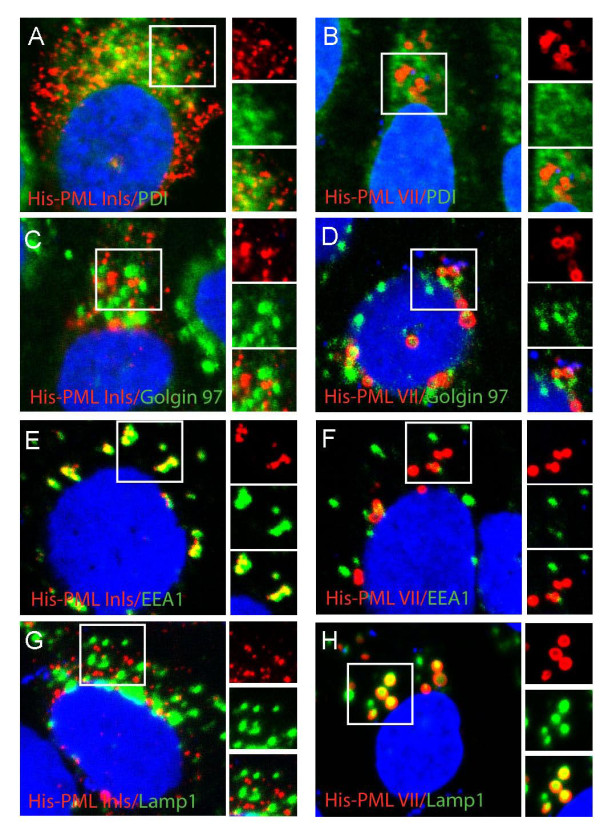
**Localization of PML to early endosomes and late endosomes/lysosomes**. U2OS cells transiently transfected with plasmids expressing His-PML Inls or His-PML VII. Cells were labelled using antibodies against His in combination with antibodies against PDI (endoplasmatic reticulum), Golgin 97 (trans-golgi network), EEA1 (early endosomes) or Lamp1 (late endosomes/lysosomes).

The experiment described above identified two distinct cytoplasmic staining patterns for PML lacking a functional NLS6: an amorphous staining pattern at peri-centriolar region defined by PML Inls and cytoplasmic vesicles targeted by PML IIInls, PML IVnls, PML Vnls and PML VII (Figure 1B and 1C). To identify these compartments, we performed double immunofluorescence labelling of His-PML Inls or His-PML VII and markers of different cytoplasmic organelles, including the endoplasmatic reticulum (labelled by anti-PDI), golgi (labelled by golgin 97), early endosomes (labelled by anti-EEA1) and late endosomes/lysosomes (labelled by anti-Lamp1). We did not detect significant co-localization between any of the PML isoforms and markers of Golgi or endoplasmatic reticulum (Figure 2A-C). His-PML Inls was, however, consistently found to overlap with or localize to the immediate vicinity of EEA1-containing early endosomes (Figure 2E). Conversely, the enlarged cytoplasmic vesicles targeted by His-PML VII did not associate significantly with early endosomes (Figure 2F), but were instead found to co-localize with Lamp1-positive late endosomes/lysosomes (Figure 2H). The cytoplasmic PML Inls was, however, not found to co-localize significantly with Lamp1 (Figure 2G). To confirm that the compartments targeted by His-PML VII were the same as those accumulating transiently expressed PML IIInls, PML IVnls and PML Vnls, we performed dual immunofluoresence labelling of Lamp1 and His-tagged PML proteins in U2OS cells transiently expressing these NLS6-defective proteins. In addition, we also performed co-transfection of these PML-expressing plasmids with a plasmid expressing GFP-Rab7, another marker of late endosomes/lysosomes. We found that PML IIInls, PML IVnls, PML Vnls and PML VII all co-localized with cytoplasmic structures containing endogenous Lamp1 and transiently expressed GFP-Rab7 (Additional file 1A). Interestingly, we noted that the Lamp1-positive endosomes, which contained PML VII or import defective PML isoforms, were enlarged compared to endosomes detected in non-transfected cells. This suggested that the relatively large size of PML-containing late endosomes/lysosomes was stimulated by PML overexpression.

### Targeting of the nuclear periphery by PML II

**Figure 3:**
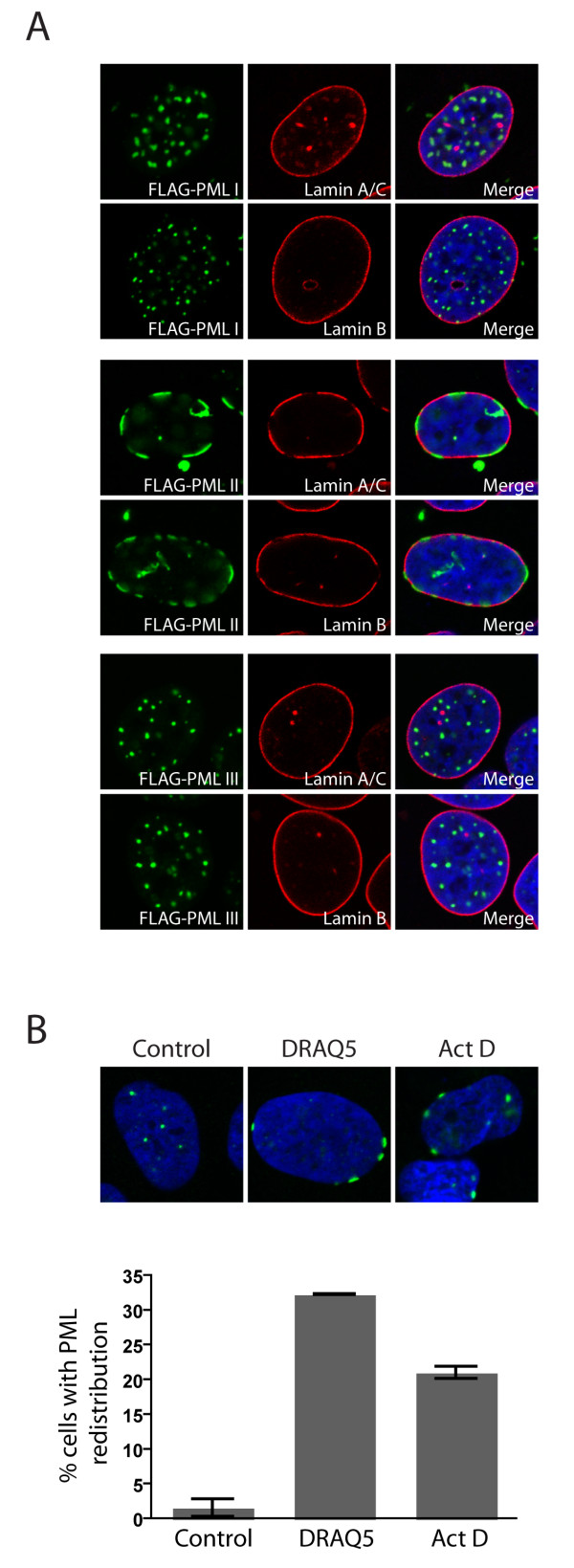
**Localization of PML II to the nuclear periphery**. A) Immunofluorescence showing FLAG-tagged PML I, II and III in lentivirus transduced U2OS cells. Overexpressed FLAG-PML II localizes to the nuclear periphery and causes exclusion of lamina. B) Relocalization of endogenous PML to the nuclear periphery in DRAQ5 or Actinomycin D treated cells. Cells were treated with DRAQ5 (2 μM) or Actinomycin D (5 μg/ml) for 2 and 4 hours respectively before fixation. Quantitation of results was performed by scoring cells with detectable PML at the nuclear periphery. The data represent the average of two independent experiments ± standard deviation. The number of cells containing rearranged PML is significantly increased in drug treated versus control treated cells (p < 0.005).

Since ectopically expressed PML II and PML IInls were found to preferentially localise to the nuclear periphery upon overexpression in U2OS cells, we wanted to determine if this PML splice variant associated with the nuclear lamina, the protein meshwork that lines the inner nuclear membrane. For these experiments we used U2OS cells that were stably transduced with a lentivirus expressing FLAG-tagged PML II. The stably transduced FLAG-PML II-expressing cells appeared to be growing normally compared to untransduced cells despite the presence of high concentrations of FLAG-PML II at the nuclear periphery. By performing immunofluorescence labelling of these cells using antibodies against the FLAG epitope in combination with anti-lamin A/C or anti lamin B, we found that PML II preferentially localized to areas of the nuclear periphery containing weak nuclear lamina staining (Figure 3A). Further, comparison of cells expressing the PML II isoform to cells expressing PML I or PML III from the same lentivirus vector, revealed that PML II (unlike PML I or PML III) induced the formation of gaps in the lamina (Figure 3A). This result suggests that PML II has the ability to alter nuclear morphology by excluding lamina from the nuclear membrane. The gaps within the lamina network formed by PML II were not caused by caspase-mediated degradation of the nuclear lamina as treatment with the caspase inhibitor Z-VAD had no inhibitory effect on their formation (data not shown). Further, immunoblots of proteins extracted from the PML II expressing cells did not reveal increased levels of caspase cleavage products relative to PML I or PML III-expressing cells (data not shown). Thus, the peripheral nuclear accumulation of PML II and concomitant formation of gaps in nuclear lamina does not appear to be related to apoptosis-induced lamina degradation. As expected, exclusion of lamina at the nuclear periphery was also observed for transiently transfected U2OS cells expressing His-tagged PML IInls (data not shown), indicating that the ability of PMLII to exclude nuclear lamina was independent of NLS6.

To determine if targeting of PML II to the nuclear periphery merely represented a phenomenon caused by PML II overexpression or if also endogenous PML has the capacity to target these nuclear structures, we examined endogenously expressed PML in U2OS cells. By immunofluorescence analysis of untransfected U2OS cells using antibodies directed against the constant part of the PML protein (thereby detecting all isoforms), we observed a small sub-fraction (between 1.5 and 2.5%) of an asynchronously growing population of U2OS cells that contained detectable PML lining the nuclear periphery. Interestingly, the number of cells containing detectable PML at nuclear membrane proximal sites increased following incubation of the cells with DRAQ5 or Actinomycin D, two reagents that are known to induce genotoxic stress (Figure 3B). This effect was, however, not observed following treatment with other genotoxic stressors/RNA synthesis inhibitors, including DRB, α-amanitin or hydroxyurea (data not shown), suggesting that recruitment of PML to the nuclear periphery is not caused by all types of genotoxic drugs or RNA synthesis inhibitors. Importantly, these results show that endogenous PML has the capacity to target the nuclear periphery and that recruitment of PML to these nuclear sites may be induced by certain types of genotoxic stress.

We also determined the ability of PML II to target the nuclear periphery in three other cell lines, including HeLa, GM847 and HaCaT. Interestingly, HaCaT and HeLa cells did not support re-localization of PML to the nuclear periphery upon PML II overexpression. Instead, these cells showed accumulation of PML II in seemingly normal PML bodies at nuclear sites distal to the nuclear periphery (Additional file 2). GM847 cells, on the other hand showed a peripheral localization of overexpressed PML II that was similar to that seen in U2OS cells (Additional file 2). Further, the two drugs DRAQ5 and Actinomycin D were found to significantly induce re-localization of PML to nuclear periphery only in U2OS and GM847 cells (the same cell lines as those supporting nuclear periphery targeting of PML II) but not in HaCaT or HeLa Cells (data not shown). This result shows that the ability of PML to target nuclear membrane proximal sites is largely cell-type dependent.

### PML II contains functional domains at the C-terminus that facilitate transport across the nuclear membrane and targeting of the nuclear periphery

**Figure 4:**
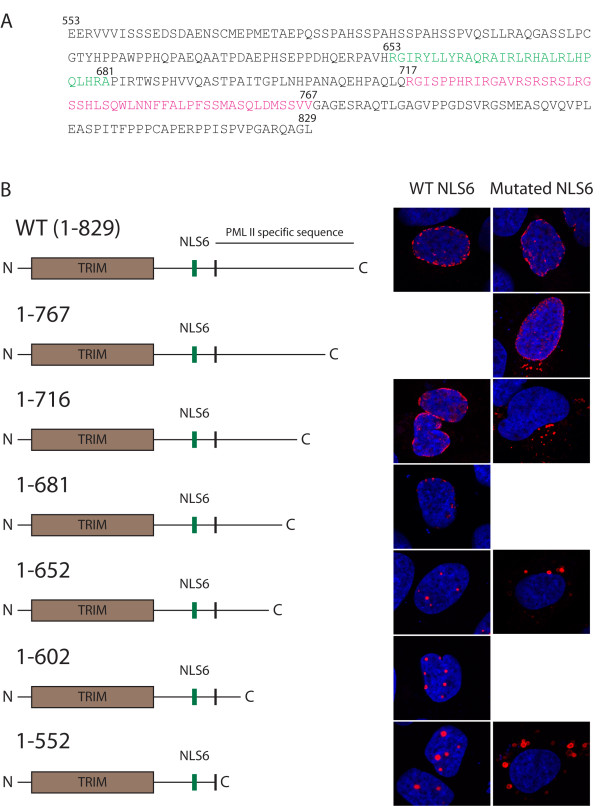
**Sub cellular distribution of PML II containing C-terminal truncations**. A) Nucleotide sequence of PML II-specific C-terminal region. The regions effecting NLS6 independent nuclear localization and targeting of nuclear periphery are highlighted in red and green, respectively. B) Subcellular distribution of His-PML II mutants in transiently transfected U2OS cells.

Since PML II is capable of both entering the nucleus independently of NLS6 as well as targeting the nuclear periphery, we wanted to determine if these two properties were induced by the same or by distinct functional domains within the PML II C-terminal variable domain. To achieve this, we constructed a series of C-terminally truncated versions of PML II and PML IInls. By transient expression of these deletion mutants in U2OS cells we found that a region between aa 653 and 681 is required for targeting of the protein to the nuclear periphery (Figure 4). Interestingly, this region overlaps the aa sequence of PML II that previously has been shown to be targeted by the Adenovirus 5-encoded protein E4 Orf3, a factor that contributes to distortion of PML NBs during the course of adenovirus infection [26]. The ability of PML IInls to migrate into the nucleus was found to be lost following deletion of aa 717-767 (Figure 4B). This region does not contain an NLS that can be predicted based on known NLSs. However, the relatively high frequency of serines and arginines within this region may suggest that this protein uses a serine-arginine-rich import signal similar to those used by a family of pre-mRNA splicing factors called SR- proteins [27-29]. Importantly, this experiment shows that distinct sequence elements within the PML II variable C-terminus are responsible for nuclear import and targeting of this protein to the nuclear periphery. This suggests the existence of at least two different functional domains that are unique for the PML II isoform.

### Targeting of early endosomes, late endosomes/lysosomes and the nuclear periphery by PML depends on a functional TRIM domain

**Figure 5:**
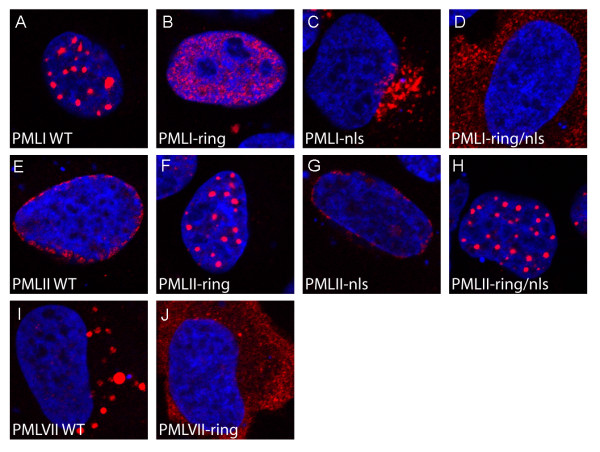
**PML clustering requires a functional TRIM domain**. U2OS cells were transfected with plasmids expressing the indicated PML isoforms and mutants. PML was visualized using an anti His antibody.

The TRIM domain has previously been shown to be important for several PML functions. In addition, this motif has been demonstrated to be important for targeting of PML to PML NBs [14]. To determine if the TRIM domain is required for directing PML to early endosomes, late endosomes/lysosomes and the nuclear periphery, we generated a set of mutated plasmid constructs expressing proteins with cysteine-to-serine mutations (C57S and C60S) in two conserved cysteines of the RING motif of PML. These conserved amino acid substitutions have been used previously to determine RING domain functions [14,30]. The RING mutation was inserted into wild type PML I, PML II and PML VII as well as in PML Inls and PML IInls. Expression of these mutants in U2OS cells revealed a clear dependence of a functional RING motif for proper subcellular targeting. PML VIIring was completely impaired in its ability to target late endosomes/lysosomes. Instead, this mutant was found to distribute diffusely throughout the cytoplasm of the transfected cell (Figure 5I-J). Similarly, PML I, which normally localizes to PML NBs, was found to distribute diffusely within the nucleus in the absence of a functional RING motif (Figure 5A-B). Further, the doubly mutated PML Inls/ring, which contains mutationally disrupted NLS6 and RING motifs, was found to distribute diffusely in the cytoplasm and was completely impaired in targeting early endosomes (Figure 5C-D). Finally, we found that PML IIring completely lost its ability to target the nuclear periphery upon overexpression in U2OS cells and was, instead, found to cluster within nuclear aggregates within the cell nucleus (Figure 5E-F). The doubly mutated PML IIring/nls protein exhibited a similar subcellular distribution as that seen for PML IIring (Figure 5G-H), confirming the ability of the PML II C-terminus to direct this protein to the nucleus in a NLS6-independent manner. The observation that PML IIring formed nuclear compartments that were reminiscent of normal PML NBs was surprising since previous studies have suggested that the formation of these nuclear structures depend on a functional RING domain. Interestingly, the structures formed by the mutated protein also recruited other PML NB resident proteins including CBP, SUMO and Daxx (Additional file 3), suggesting that they may have some features in common with normal PML NBs.

## Discussion

The present study identifies early endosomes, late endosomes/lysosomes and structures at the inner nuclear membrane as targets for PML isoforms lacking a functional NLS6 (Table 1). The selection of target organelles by each of the isoforms appears to be regulated by their C-terminal variable domain. In addition, the ability of the various PML isoforms to sequester at their respective cellular sites is found to be largely dependent on the presence of a functional TRIM domain. Thus, distinct functional elements at both the N-terminal as well as the C-terminal portion of PML may cooperate to achieve correct subcellular localization of the protein. Such dual requirement for functions at the N-terminus and C-terminus for proper protein localization may represent a general trait of TRIM family proteins. This is suggested by a general ability of several proteins containing a TRIM domain to cluster at specific cellular sites and by the conserved organization of this family of proteins into a constant N-terminal region (containing the TRIM domain) and a flexible C-terminal tail that may contain different types of functional domains.

**Table 1:** Target compartments of wild-type and nuclear import-defective PML isoforms in U2OS cells

***PML isoform***	***Wild type***	***Nuclear import-defective mutant***
PML I	PML NBs	Early endosomes
PML II	Nuclear periphery	Nuclear periphery
PML III	PML NBs	Late endosomes/lysosomes
PML IV	PML NBs	Late endosomes/lysosomes
PML V	PML NBs	Late endosomes/lysosomes
PML VII	Late endosomes/lysosomes	Not applicable

The ability of PML to target early and late endosomes may reflect a role for PML in endosome trafficking. A functional association of PML with early endosomes has previously been reported for a splice variant of PML that lacks exon 5 and 6 and that contains an N-terminal configuration similar to that of PML IV. In this case, cytoplasmic PML was found to function in TGFβ-mediated signaling through interactions with SMAD1, SMAD2 and SARA at early endosomes [23]. Further, since several viruses and bacteria are known to exploit endosomal trafficking routs as a means to invade their host, the ability of PML to target these cytoplasmic organelles may also reflect a role of this protein in the cellular defense against pathogens. In agreement with this, PML represents an interferon responsive gene (which is characteristic for genes involved in the innate immune response) and has been shown to restrict replication of certain viruses [3,31,32]. In a recent study, production of splice variants of PML lacking exon 5 and 6 was shown to be increased in interferon treated and HSV1-infected cells [24]. This finding suggests the existence of a regulatory mechanism whereby cells respond to virus infection by altering the splice pattern of PML to obtain increased expression of cytoplasmic versus nuclear PML. The present study indicates that PML proteins produced by mRNA species lacking exon 5 and 6 will be expected to target late endosomes/lysosomes. Further studies are needed to determine if PML exerts its antiviral property by interfering with endosomal or lysosomal functions.

A limitation of the present study is that we were unsuccessful in detecting endogenous PML within early or late endosomes by immunofluorescence labeling using anti-PML antibodies. In fact, PML in most types of cultured mammalian cells are primarily detected within PML NBs where it is most highly concentrated. We cannot fully exclude the possibility that the ectopically expressed NLS6-defective PML detected in endosomes represents protein aggregates that are in the process of being cleared from the cell by lysosome-mediated degradation. However, several observations suggest a functional role of PML at these locations. First, the observed subcellular distribution of PML to late endosomes is dependent on a functional RING motif. This suggests that a functional TRIM is required for endosome targeting. Second, the cytoplasmic staining of PMLVII as well as PML IIInls, PML IVnls and PML Vnls seem to be present mostly at the exterior of these organelles and not at their interior as would be expected if PML was engulfed by lysosomes. Third, the PML isoforms expressed in the cytoplasm enhances the size of late endosomes/lysosomes, suggesting a stimulatory role of cytoplasmic PML on these compartments. Lastly, overexpressed NLS6-proficient PML proteins can also be observed to form aggregates in the cytoplasm that are readily detected by immunofluorescence, but for these proteins we have never observed co-localization with the lysosomal marker proteins Lamp1 or Rab7. Thus, NLS6 may have a direct role in preventing PML isoforms that are destined to target the nucleus from interacting with late endosome/lysosomes. The PML protein was recently identified in a proteomic screen for phagosomes resident proteins [33]. Thus, even though PML generally is undetectable by immunofluorescence in most cytoplasmic compartments, this protein may be present in organelles such as endosomes and phagosomes at levels that are undetectable by antibodies that we have available.

PMLII overexpressed in U2OS cells was found to primarily target the inner nuclear membrane. Distribution of PMLII to these nuclear sites was also noted in a previous study following expression of this isoform in Chinese hamster ovary cells [34]. This particular distribution of PML appears to be highly cell type specific as PMLII expressed in HaCaT or HeLa cells exhibited PML clusters at more central regions of the nucleus consistent with normal PML NB morphology. Interestingly, both cell lines (U2OS and GM847) that were found to support targeting of PML to the nuclear periphery are ALT cells, cells that use alternative lengthening of telomeres (ALT) and not telomerase as their primary mechanism for telomere elongation [35,36]. It has previously been shown that ALT cells contain special PML NBs termed ALT associated PML bodies (APBs) that sequester DNA repair proteins and telomeric DNA [37]. Thus, the ability to direct PML to the nuclear periphery may represent an additional phenotype that accompanies ALT cells.

So far we have not been able to detect structural components of the nuclear periphery that co-localized with PML at the edge of the nucleus. In fact, both nuclear lamina as well as nuclear pore complexes (as detected with the nucleoporin-specific antibody Mab414; data not shown) were found to be excluded from sites containing overexpressed PMLII. The association of PML with the nuclear periphery may reflect a role of this protein in cellular processes such as transcription regulation, DNA replication or DNA repair since these cellular processes are known to be active at nuclear envelope proximal sites [38-40]. Interestingly, the region of PMLII that we found to be responsible for inducing nuclear periphery targeting overlaps the amino acid sequences previously shown to interact directly with the adenovirus protein E4 Orf3, which is known to be involved in PML NB disruption during adenovirus infection [26]. This may indicate that the activity supported by PMLII at the nuclear periphery represents a barrier that certain viruses need to overcome in order to achieve successful infection.

The presence of an extra NLS at the C-terminal variable domain of PMLII suggests that some splice variants of this protein may enter the nucleus even in the absence of amino acid sequences encoded by exon 6. Analysis of the C-terminal region of PMLII does not reveal peptide sequences that match the consensus sequence of any known NLSs [41]. However, the region between aa 717 to 767, which in the present study was shown to be important for import activity, is rich in arginines and serines. This may suggest the presence of a serine arginine-rich NLS similar to that used by SR-proteins, a group of proteins involved in pre-mRNA splicing [27-29]. The lack of sequence similarity between NLS6 and the NLS present within PMLII C-terminal variable region, suggests that PML II uses two distinct nuclear import routes.

NLS6 seems to represent an unconventional NLS that may play a central regulatory role in several aspects of PML trafficking and subcellular localization. Besides playing an important role in nuclear import, this peptide sequence has also been shown to be required for targeting PML to cytoplasmic PML-containing compartments referred to as cytoplasmic assemblies of PML and nucleoporins (CyPNs) [30]. These structures form during the mitosis-to-G1 transition of the cell cycle and seem to be derived directly from post mitotic PML NBs [30]. NLS6 may also be regulated by SUMOylation since this peptide sequence is known to contain one of the three lysine residues that represent SUMO conjugation sites in PML. However, abrogation of this SUMOylation site by mutagenesis was found not to affect PML nuclear import, suggesting a role of this SUMOylation event that is not directly related to import [22]. Finally, genome sequencing analysis of patients with an aggressive ATRA-resistant form of APL revealed mutations in the non-rearranged PML gene that is predicted to cause premature translation termination of the PML protein upstream of NLS6 [42]. Based on the data presented in the present study, such mutants may contribute to tumorigenesis through interference with late endosomes/lysosomes functions.

## Conclusions

In this study we have shown that forced expression of PML in the cytoplasm leads to clustering of this protein at different types of cellular compartments, including early endosomes, late endosomes and the inner nuclear membrane. Since the ability of PML to cluster at each of these different sites depends on a functional TRIM domain, our data support the notion that the TRIM domain plays a general role in protein clustering and that the alternatively spliced C-terminus of the protein has a specific role in compartment selection. Further studies are needed to elucidate potential functions of PML at early endosomes, late endosomes and the nuclear periphery.

## Methods

### Cell-lines and transfection

U2OS cells (human osteosarcoma), GM-847 cells (SV40 transformed human fibroblasts), HaCaT (human keratinocyte) and HeLa (human cervical cancer) were maintained in Iscove's modified Dulbecco's medium (IMDM; Bio Whittaker, Belgium) containing 10% foetal calf serum (PAA, Austria) at 37°C and 5% CO2. Cells were transfected using the FuGENE6 transfection reagent (Roche, Switzerland) according to the manufacturer's protocol. U2OS cells stably expressing Flag-tagged PML I, II or III were generated using lentivirus constructs generously provided by Dr Roger D Everett at MRC virology unit, Glasgow, UK.

### Plasmid constructs

His-tagged PML I through V expressed from a pcDNA3 vector were kindly provided by Dr. K-S Chang at the University of Texas, Austin, Texas [21]. His-tagged PML VII and NLS mutants (K486A_K487A) of PML I through V were described in [30]. The PML II truncation mutants were made by PCR cloning using the forward primer (gaagcccagcctatggctgtg) in combination with the reverse primers (tatgaattcatgcctccccggcgccactggc (1-552), tatgaattcaagactggactggcgaggagtg (1-602), tatgaattcagtggacggcagggcgctc (1-652), tatgaattcattgcagctgggcaggatgttc (1-716) or tatgaattcacaccacggaagacatgtcaag (1-767)). The PCR product was then substituted for the PML II specific sequence of pcDNA3 His-PML II or pcDNA3 His-PML IInls using the Van91I (Fermentas, Canada) and Eco RI (NEB, MA) restriction sites. The 1-681 mutant was constructed by digesting the pcDNA3 His-PML II vector with Apa I restriction enzyme (Takara, Japan) and re-ligation. This resulted in the loss of PML II aa 682-829 and formation of a short, 12 aa, nonspecific tail at the end of the protein.

RING finger mutants of PML I, II and VII were constructed by introducing point-mutations (C57S;C60S) to the respective His-tagged PML isoforms using the QuikChange kit (Stratagene, CA). The His-PML I-RN and His-PMLII-RN, RING finger and NLS double mutants, were constructed by introducing the RING finger and NLS mutations in two subsequent reactions using the QuikChange kit. The plasmid expressing GFP-tagged Rab7 was kindly provided by Dr. Harald Stenmark at Rikshospitalet, Oslo, Norway.

### Immunofluorescence

Cell fixation and immunofluorescence labeling was performed as described previously [43]. Primary antibodies used were mouse anti-His (HIS-H8), mouse anti-PML (PG-M3), rabbit anti-PML (H238) mouse anti-Lamp1 (H5G11) and mouse anti-Lamin A/C (636) (all from Santa Cruz, CA), rabbit anti-Lamin B1 (Abcam, UK) and mouse anti-Flag (F3165) (Sigma, MO). Secondary antibodies were FITC or Texas-Red labeled goat antibodies against mouse immunoglobulin subtypes or against rabbit antisera (Southern Biotech, AL).

Microscopic images were obtained using a Zeiss LSM510 Meta laser confocal microscope (Zeiss, Germany) with a 63× oil immersion lens (Zeiss).

### Chemicals and treatment

DRAQ5 treatment was performed by adding DRAQ5 (Biostatus, UK) to a final concentration of 2 μM, to the medium for two hours before fixation. Actinomycin D treatment was performed by adding Actinomycin D (Sigma), to a final concentration of 5 μg/ml, for four hours before fixation. To quantify cells with PML at the nuclear periphery, cells were counted manually in the microscope. For each sample, four hundred cells were counted and scored for the presence of PML staining at the nuclear periphery. In each case, two independent parallels were counted.

## Authors' contributions

ÅJL carried out the majority of the experiments. AG prepared some of the PML mutants and performed some of the experimental work. SOB designed and coordinated the study. ÅJL drafted the manuscript and SOB wrote the manuscript with assistance from ÅJL and RB. All authors red and approved the final manuscript.

## Supplementary Material

Additional file 1 **Co-localization of PML to late endosomes/lysosomes**. A) Immunofluorescence labelling showing co-localization of His-tagged PML VII with Lamp1 (left panels) or transiently expressed YFP-Rab7 (right panels). B) Immunofluorescence images showing co-localization between nuclear import-defective PML isoforms and Lamp1. Click here for file

Additional file 2 **PML II distribution in different cell lines**. U2OS and HaCaT cells were stably transduced using a lentivirus expressing FLAG-tagged PML II. GM847 and HeLa cells were transiently transfected by a plasmid expressing His-tagged PML II. Click here for file

Additional file 3 **Co-localization of His-PML II, His-PML IIring and His-PML IInls with PML NB resident proteins**. U2OS cells were transfected with plasmids expressing wt or mutated PML II and subsequently immunolabeled using an anti His antibody in combination with antibodies targeting the indicated proteins. Click here for file
