# Supplementary figures and images for: Subcellular distribution of nuclear import-defective isoforms of the promyelocytic leukemia protein
Source: BMC Mol Biol. 2010 Nov 21;11:89. doi: 10.1186/1471-2199-11-89 (PMC2998510; doi:10.1186/1471-2199-11-89)

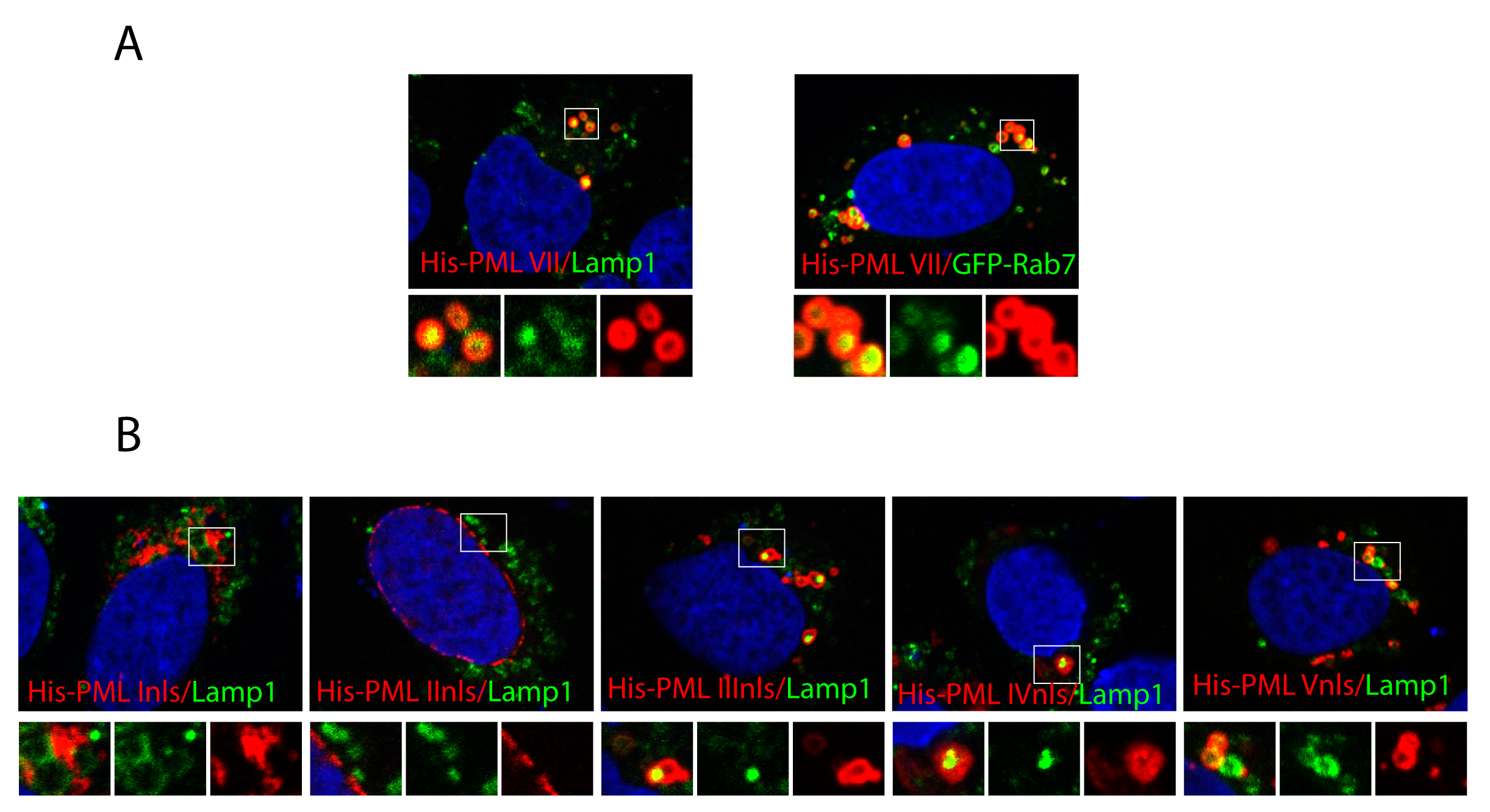

Supplement: Additional file 1 — Co-localization of PML to late endosomes/lysosomes. A) Immunofluorescence labelling showing co-localization of His-tagged PML VII with Lamp1 (left panels) or transiently expressed YFP-Rab7 (right panels). B) Immunofluorescence images showing co-localization between nuclear import-defective PML isoforms and Lamp1. [file 1471-2199-11-89-S1.TIFF]

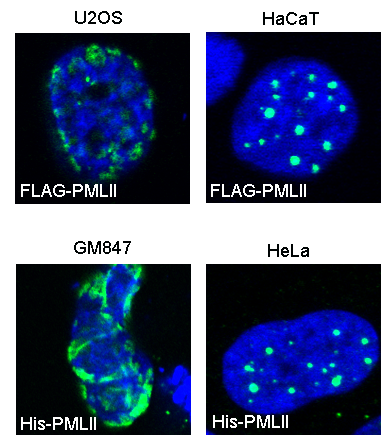

Supplement: Additional file 2 — PML II distribution in different cell lines. U2OS and HaCaT cells were stably transduced using a lentivirus expressing FLAG-tagged PML II. GM847 and HeLa cells were transiently transfected by a plasmid expressing His-tagged PML II. [file 1471-2199-11-89-S2.TIFF]

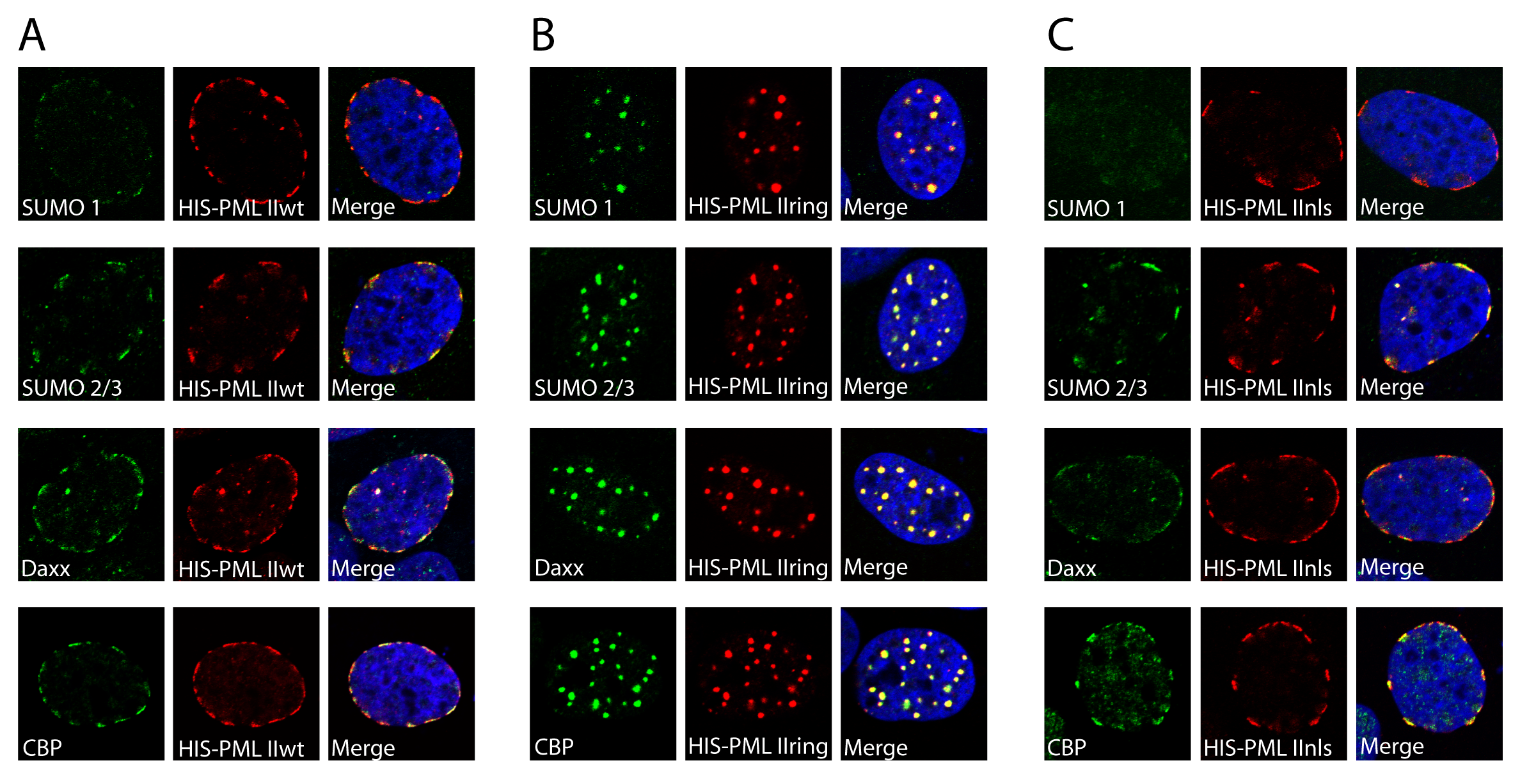

Supplement: Additional file 3 — Co-localization of His-PML II, His-PML IIring and His-PML IInls with PML NB resident proteins. U2OS cells were transfected with plasmids expressing wt or mutated PML II and subsequently immunolabeled using an anti His antibody in combination with antibodies targeting the indicated proteins. [file 1471-2199-11-89-S3.TIFF]
